# Phase 1 Evaluation of MF-300: An Investigational First-in-Class Oral Candidate for Sarcopenia

**DOI:** 10.1093/geroni/igaf122.3994

**Published:** 2025-12-31

**Authors:** Leigh MacConell, Amrik Shah, Diane Mould, Siva Lavu, Micah Webster

**Affiliations:** Alchemeos Clinical Consulting, Encinitas, California, United States; Karma Statistics LLC, Skillman, New Jersey, United States; Projections Research Inc, Phoenixville, Pennsylvania, United States; RedOwlet LLC, Wellesley, Massachusetts, United States; Epirium Bio, San Diego, California, United States

## Abstract

Age-related sarcopenia, a progressive chronic disease involving loss of skeletal muscle mass and function, contributes to disability and loss of independence, affecting 1/3 of Americans over 60 years. Despite being associated with increased risk of falls, hospitalization, and all-cause mortality, no pharmacologic treatment is approved for sarcopenia, representing an important unmet medical need. MF-300 is an investigational, first-in-class, orally administered, 15-hydroxyprostaglandin dehydrogenase inhibitor that improves aged muscle force in preclinical studies and is in development for sarcopenia. Safety, tolerability, pharmacokinetics (PK) and pharmacodynamics (PD) of MF-300 were evaluated in healthy adults in a Phase 1 double-blind Single Ascending Dose (SAD) and Multiple Ascending Dose (MAD) study. In the SAD, MF-300 or placebo were administered to 40 subjects at 5 doses (75mg to 800mg). In the MAD, dose escalation included QD doses of 75mg, 125mg, and 200mg for 5 days to 30 subjects. Urinary prostaglandin E2 and its metabolites were investigated as MF-300 target engagement biomarkers. All participants completed the study. MF-300 was observed to be safe and well-tolerated; all but one adverse event was mild with no dose relationship. There were no serious adverse events. The PK profile showed rapid absorption (Tmax 1.17h-2.58h) and a T1/2 supporting QD dosing (12.2h-15.9h). Exposure metrics (Cmax, AUC) increased slightly greater than dose-proportionally and were consistent with anticipated therapeutic activity based on preclinical data. Dose-related changes in PD biomarkers were consistent with MF-300 target engagement. These findings support further Phase 2 evaluation of MF-300 in age-related sarcopenia.

